# Deep learning reveals what facial expressions mean to people in different cultures

**DOI:** 10.1016/j.isci.2024.109175

**Published:** 2024-02-10

**Authors:** Jeffrey A. Brooks, Lauren Kim, Michael Opara, Dacher Keltner, Xia Fang, Maria Monroy, Rebecca Corona, Panagiotis Tzirakis, Alice Baird, Jacob Metrick, Nolawi Taddesse, Kiflom Zegeye, Alan S. Cowen

**Affiliations:** 1 Research Division, Hume AI, New York, NY 10010, USA; 2 Department of Psychology, University of California, Berkeley, Berkeley, CA 94720, USA; 3 Department of Psychology and Behavioral Sciences, Zhejiang University, Hangzhou, Zhejiang, China; 4 Node Survey Solutions, Addis Ababa, Ethiopia

**Keywords:** Artificial intelligence, Psychology, Social sciences

## Abstract

Cross-cultural studies of the meaning of facial expressions have largely focused on judgments of small sets of stereotypical images by small numbers of people. Here, we used large-scale data collection and machine learning to map what facial expressions convey in six countries. Using a mimicry paradigm, 5,833 participants formed facial expressions found in 4,659 naturalistic images, resulting in 423,193 participant-generated facial expressions. In their own language, participants also rated each expression in terms of 48 emotions and mental states. A deep neural network tasked with predicting the culture-specific meanings people attributed to facial movements while ignoring physical appearance and context discovered 28 distinct dimensions of facial expression, with 21 dimensions showing strong evidence of universality and the remainder showing varying degrees of cultural specificity. These results capture the underlying dimensions of the meanings of facial expressions within and across cultures in unprecedented detail.

## Highlights

• We trained a deep neural network to predict the culture-specific meanings of facial expressions
• We gathered 423,193 facial expressions from 5,833 participants in 6 countries
• Participants rated each expression in their own language in terms of 48 emotions
• We uncovered 28 distinct dimensions of facial expression, with 21 showing cultural universality

## Graphical abstract

**Figure:**
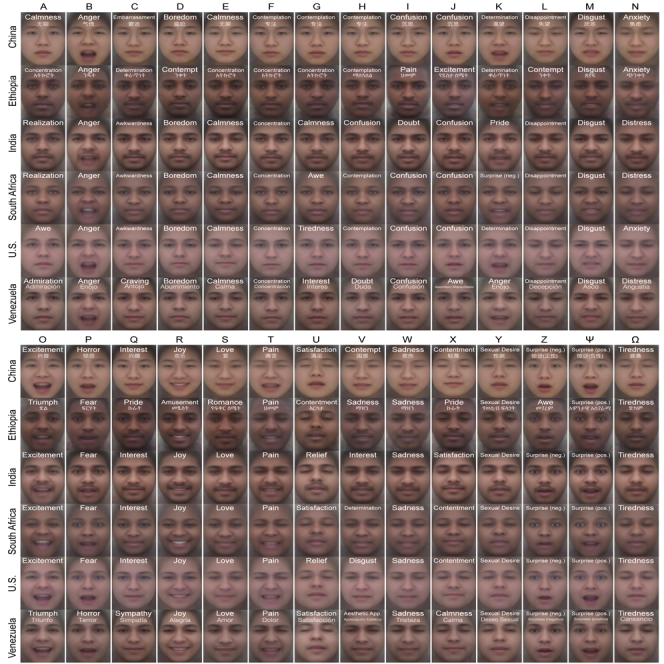

## Introduction

Humans effortlessly infer meaning from facial expressions: a smiling person looks happy the way an apple looks red. But philosophers and scientists have debated the meaning of facial expressions for centuries.^1^^,^^2^^,^^3^^,^^4^^,^^5^^,^^6^^,^^7^ Darwin theorized that dozens of facial expressions had evolutionary underpinnings.^1^ Paul Ekman posited six universal facial expressions—of anger, disgust, fear, happiness, sadness, and surprise^3^—but expressed an openness to many other universal and culture-specific expressions.^8^ Others have proposed that facial expressions convey pleasantness or unpleasantness, arousal or calmness, and features of situational appraisal or motivation,^5^^,^^9^^,^^10^ which recent studies have found can be concisely summarized in terms of emotion concepts (including nuanced states such as doubt and sympathy^7^^,^^11^).

Methodologically, however, studies have largely focused on how images of the six facial expressions focal to Ekman’s early work are described by small samples of participants in one or two cultures. Such studies have limitations. Foremost among them is sample size: due to statistical limitations, small samples are biased toward low-dimensional taxonomies of emotional expression (such as two-dimensional valence and arousal models) even if the underlying dimensionality of the data is high.^12^ In addition, they are subject to perceptual and linguistic biases. The perception of a person’s facial movements can be confounded by their physical appearance—including factors such as gender, age, and face shape—and context.^13^^,^^14^^,^^15^^,^^16^^,^^17^ Moreover, emotion concepts do not always have perfect translations across languages.^18^ Existing studies, while providing a valuable foundation for our understanding of facial expressions, have been underpowered to fully account for these various influences on the inferred meanings of facial expressions.

Thus, a more generalized understanding of the meanings of facial movements within and across cultures is still only beginning to emerge. There is broad consensus that “the face is a rich source of information.”^6^^,^^7^ But to determine what information the face conveys, it will be necessary to move beyond traditional methods such as small samples of participants and stimuli with predefined discrete meanings.

Such methods have recently been introduced to the study of facial expression, with results suggesting that facial expressions are high-dimensional and unlikely to reduce to the smaller taxonomies that have been traditionally assumed. A study of hundreds of thousands of judgments of 1,500 naturalistic facial expressions revealed that the perception of facial expression is much more complex than previously assumed, with at least 28 distinct dimensions of meaning blending a wide range of emotions (“awe,” “concentration,” “sympathy”^7^). A large-scale study of expressions used web search engines to discover that dozens of configurations of 14 facial muscle movements can reliably be found alongside emotion and affect terms in five languages.^19^ Another study used machine learning to examine the actual contexts in which 16 broader dimensions of facial expression occur in millions of natural videos, finding substantial similarity in the social contexts in which specific expressions were observed worldwide.^11^ Large-scale, data-driven studies converge in showing that naturally occurring facial expressions are fundamentally high-dimensional, in contrast to the low-dimensional theory-driven explanations of earlier work.

However, studies of naturally occurring images of facial expressions, even at a large scale, also have important limitations. Critically, they provide no direct insight into what facial expressions mean to the people making them (who generally cannot be reached to provide self-report data). Additionally, studies that rely upon perceptual judgments of natural images are potentially confounded by the physical appearance and context of the person making the expression.^7^^,^^19^ In existing studies where machine learning is used to rule out such influence,^11^^,^^19^ the algorithms were trained prior to the study on perceptual ratings of facial expressions in a single culture, using predetermined taxonomies of expression, capturing less than half of the variance in facial expression in the culture where they were trained. Many of the outputs of these algorithms are confounded by perceptual biases in the data they are trained on that have to be discarded.^11^ Thus, several fundamental questions about facial expressions remain unanswered. How many distinct meanings do facial expressions convey? How can these meanings be precisely conceptualized? How well are they preserved across cultures?

In the present investigation, we perform a large-scale, experimentally controlled study of what facial expressions mean to the people making them in six countries, and generate data that is suitable for both machine learning and psychological inference. This allows us to use deeply inductive methods to characterize the distinct cross-cultural and culture-specific meanings of a rich space of facial expressions. We devised an experimentally controlled task to generate a wide range of facial expressions that would enable us to control for the confounding influence of identity, demographics, and context in our analyses. Starting with 4,659 wide-ranging naturalistic images, we had globally diverse participants photograph themselves mimicking the facial expressions in the images, resulting in 423,193 participant-contributed facial expressions. Since the identity and demographics of the individuals in the mimicked images were experimentally randomized while maintaining the same underlying expressions, the mimicry procedure allowed us to decouple ratings of the expressions from these confounding influences. We explored a wide range of meanings of the mimicked facial expressions by gathering intensity ratings corresponding to 48 emotion and mental state concepts, in six countries and four languages. Intensity was rated on a scale from 0 to 100, as in many other studies on the perceived meanings of facial expressions.^20^^,^^21^^,^^22^^,^^23^ Finally, without assuming any correspondence in ratings across countries, we mapped the dimensions of emotional meaning to underlying visual dimensions with machine learning.

The 48 emotions and mental states used to rate each facial expression were derived from a comprehensive examination of the meanings facial expressions have been previously posited to convey, and the words that people regularly use to describe emotion-related experiences and expressions. It is important to note that the purpose of this study is to determine what facial expressions convey to perceivers: that is, regardless of the underlying experiences of the people in the images, our experiment was designed to determine what facial actions convey externally. The purpose of our study is not to determine which facial actions provide definitive evidence of an underlying emotional experience.

Importantly, we controlled for the influence of the specific demographics of the individuals in the seed images by training a deep neural network (DNN) to predict the average meanings inferred from facial expressions solely from mimicked photographs by globally diverse individuals. This approach allowed us to model cultural differences and avoid mistranslating emotion concepts across cultures by training the DNN to predict average meanings in each country separately. This means that the DNN had no prior knowledge of how emotion concepts translated across cultures or languages (a facial expression found to be associated with “joy” in one country could still just as easily be found to be associated with “sadness” in another, even if they speak the same language). Finally, we assessed how many distinct dimensions of meaning were captured by the DNN using principal preserved components analysis (PPCA). The present research thereby documents the ways that dimensions of facial expression overlap, diverge, and blend across a wide range of modern cultures that vary in terms of self-construals, values, and patterns of social and economic organization.

To derive a cross-cultural dimensional structure of the meaning of facial expressions using methods unaffected by perceptual, linguistic, and demographic biases, we conducted a large-scale experiment in two phases. In the first phase of data collection (henceforth “mimicry phase”), a total of 5,833 participants from China (n = 602; 371 female), Ethiopia (n = 149; 26 female), India (n = 478, 74 female), South Africa (n = 2,131; 970 female), the United States (n = 2,576; 1,346 female), and Venezuela (n = 344; 110 female) completed a facial expression mimicry task, imitating randomly sampled subsets of 4,659 images of facial expressions (see STAR Methods) and rating what each expression meant to them as they were imitating it (see Table S1; Figure S1 for extended demographic information). All participants provided informed consent and all aspects of the study design and procedure were approved by Heartland IRB (HIRB project no. 031221–315).

These six countries are widely diverse in terms of culture-related values of interest in cross-cultural comparisons: for example, they cover a wide range in terms of dimensions such as individualism vs. collectivism (the United States consistently ranks high in individualism; Venezuela, Ethiopia, and China are high in collectivism; and the other countries fall closer to the middle of the distribution^24^), and power distance (Venezuela and China rank high in power distance, the United States and South Africa are lower in power distance, and Ethiopia and India fall closer to the middle of the distribution.^24^^,^^25^)

The seed images were 4,659 images of people’s facial expressions, extracted from naturalistic datasets of emotional stimuli,^26^ expressive behavior found online using hundreds of search queries for emotion concepts and emotional contexts,^7^ and responses to 1707 emotionally evocative films^27^ (see STAR Methods for breakdown). Based on past estimates of the reliability of observer judgments of facial expressions,^7^ we collected responses to each seed image from an average of 15.2 separate participants in each culture. This amounted to a total of 423,193 experimental trials with associated mimic images and judgments on the meaning of the expression.

Prior to engaging in the mimicry task, participants were instructed to use their computer webcam to photograph themselves on each trial. On each trial, participants saw a target facial expression and were instructed to mimic the expression in the image such that their imitation would be rated similarly to the original image. This paradigm leverages the ability of most humans to mimic facial expressions (facial mimicry), which is observed early in development and often occurs spontaneously during social interaction.^28^^,^^29^^,^^30^ Participants completed 30 trials per survey and could complete multiple versions of the survey, up to ten depending on the country.

On the same survey page, participants were asked to judge what they thought the person was feeling by selecting from 48 terms for emotions and mental states and then rating each selection from 0 to 100, with values reflecting the perceived intensity of the emotion. Participants were required to select a value on a rating scale for at least one category (See Figure S2 for distributions of ratings for seed images). English terms were used in the three out of six countries where English is an official language (India, South Africa, and the United States). In China, ratings were collected in Chinese; in Ethiopia, ratings were collected in Amharic; and in Venezuela, ratings were collected in Spanish (see Table S2 for a complete list of terms and their translations in each language). This mimicry phase of data collection resulted in a large number of participant-generated “mimic” images in each culture [China (n = 60,498), Ethiopia (n = 29,773), India (n = 58,054), South Africa (n = 107,364), the United States (n = 170,013), and Venezuela (n = 47,734)] and self-report ratings corresponding to each mimic image that can simultaneously be considered perceptual ratings of each seed image. While posed, the mimic images are organized according to granular self-report judgments of the emotions the person in the image believes others will perceive, rather than experimenter- or theory-driven assumptions.

We made further use of these stimuli in the second phase of data collection (henceforth “rating-only phase”), in which we recruited an independent set of participants from each culture [China (n = 542; 349 female), Ethiopia (n = 78; 18 female), India (n = 1,101; 507 female), South Africa (n = 2,465; 1,565 female), the United States (n = 3,419; 1,983 female), and Venezuela (n = 352; 118 female)] to complete an emotion perception task in which they rated mimic images from the first phase of data collection that were generated by participants within their same country. Participants’ ratings in this task thus capture culture-specific understandings of the original seed stimuli but, unlike the self-report ratings from the mimicry task, could not have been influenced by the perception of any demographic or contextual influences on the visual properties of the seed stimuli. Thus, their correlations with the seed stimuli represent measures of the emotional meaning of these original expressions that are invariant to certain perceptual biases and confounds (gender, age, race) that can be problematic for studies of facial expression. As in the mimicry phase of data collection, participants were asked to judge each face along 48 terms for emotions and mental states and provide intensity ratings ranging from 0 to 100. On average, participants in this phase of the experiment completed 77.1 trials. This amounted to a total of 534,459 judgments of all mimic images (see Figure S2 for distributions of emotion ratings for mimic images).

## Results

### Shared dimensions of facial emotion perception

In our recent work on computing shared dimensions of emotional experience and expression between two cultures, we used a method we are calling principal preserved components analysis (PPCA^31^^,^^32^^,^^33^^,^^34^). In the present study, with datasets measuring the same attributes in 6 different countries, we developed a generalized version of the PPCA algorithm (G-PPCA) that extracts linear combinations of attributes that maximally co-vary across three or more datasets (in this case, emotion judgments from 6 countries). The resulting components are ordered in terms of their level of positive covariance across all 6 datasets (see STAR Methods for more information on the method and significance testing).

We first investigated the cross-cultural dimensions of perceived emotion using judgments of the seed images collected during the mimicry phase of data collection. We applied G-PPCA to judgments of the 4,659 seed images across the 6 countries. We found that 31 semantic dimensions, or distinct kinds of emotion, were preserved across all six cultures in emotion judgments of the seed images, providing an initial estimate of how many distinct emotions are perceived similarly across cultures (Figure 2; see Figure S3 for G-PPCA results across judgments of both seed and mimic images). This extends prior work showing that perceivers reliably distinguish at least 28 dimensions of meaning in facial expression within a culture^7^ and that a high-dimensional semantic space organizes other components of emotional experience and perception across cultures.^32^^,^^35^^,^^36^ This work also converges with the high dimensional structure of emotion observed in more top down studies of emotion production and recognition.^37^^,^^38^^,^^39^^,^^40^

However, despite the scale of the dataset, the findings from this first analysis could be explained in part by contextual influences on emotion perception such as visual context and demographic factors such as gender and culture. In particular, the ratings that went into this analysis could be influenced by the subtle contexts visible in the periphery of each image as well as the demographic characteristics of the individuals forming expressions in the images, rather than just the information conveyed by the underlying facial movements.

To control for the effects of visual information beyond facial movement, we trained a deep neural network (DNN) to predict the meanings attributed to facial movements while ignoring physical appearance and context. This allowed us to derive a structural taxonomy of expressive facial movement within and across cultures.

### Culture-specific emotion regression using deep neural networks

DNNs are powerful machine learning algorithms that can accomplish many real-world tasks. Given that recent advances in computing power have allowed DNNs to approximate or exceed human performance in domains such as classifying images or translating text, DNNs have also emerged as scientifically useful models for understanding the psychological processes involved in these domains (e.g., object recognition^41^^,^^42^^,^^43^). Specifically, they provide a mathematical model of human perceptual judgments that are directly tethered to the sensory input.

**Figure 1:**
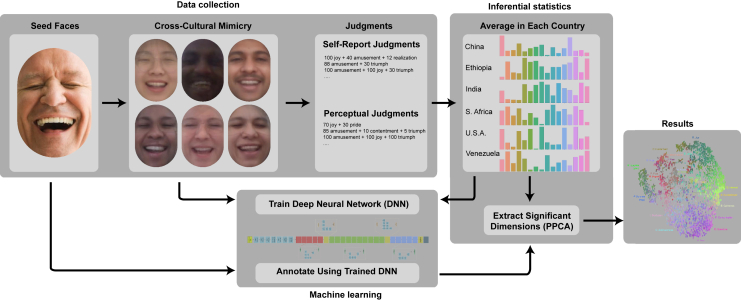
Schematic of our experimental and analytic approach An initial set of 4,659 seed images were rated by 5,833 participants in 6 countries. These participants also used a computer webcam to photograph themselves mimicking subsets of seed images. Thus, each of the resulting 423,193 mimic images had a corresponding self-report judgment reflecting what the facial expression meant to the person making it. An additional 7,957 participants provided perceptual judgments of mimic images produced in their own country. We used a deep neural network (DNN) to find dimensions of facial expression that had distinct meanings within or across cultures, independent of facial appearance and context, by averaging all self-report and perceptual ratings corresponding to each seed image in each country and tasking the DNN with predicting these averages from the mimic images alone (where the facial appearance and context were decorrelated with the corresponding properties of the seed image via experimental randomization). Finally, we evaluated the DNN on the original seed faces (to which it had no exposure during training) and compared these predictions to the average judgments in each country to extract the dimensions of meaning that the DNN significantly predicted from facial movement alone.

We tasked a DNN to predict the average emotion judgments of each seed image in each culture from the images of participants mimicking each seed image (see Figure 1). Because the seed images were each shown to a random set of participants, this method forced the DNN to remain invariant to the physical appearance and context of the person making the expression (factors such as gender, age, clothing, and lighting that were randomized relative to the expression being imitated). Further, we treated the average emotion judgments within each culture (made in four separate languages) as separate outputs. Thus, the DNN was not provided any prior mapping between emotion concepts and their use across countries or attempted translations across languages (English, Amharic, Chinese, and Spanish). (We adapted our DNN architecture from the FaceNet Inception Resnet v1 model,^44^ pretrained on the VGGFace2 dataset using transfer learning.^45^^,^^46^ We used MTCNN to extract the faces from each mimic image,^47^ so only the face was used as input to the model. For further details on model architecture and training, see STAR Methods).

After training, we applied the DNN to the seed images (to which it was not exposed during training). Finally, we applied a multidimensional reliability analysis method to distill the significant shared and culture-specific dimensions of facial expression uncovered by the DNN. Specifically, we applied principal preserved components analysis (PPCA) between the DNN’s culture-specific annotations of the seed images and the emotions inferred from the seed images by participants in each culture. Given that no prior was built into the model linking the words from different languages to one another, any relationship uncovered between the emotion concepts across languages using this method implies that the concepts were used similarly to describe the same facial movements (see Figure 1).

**Figure 2:**
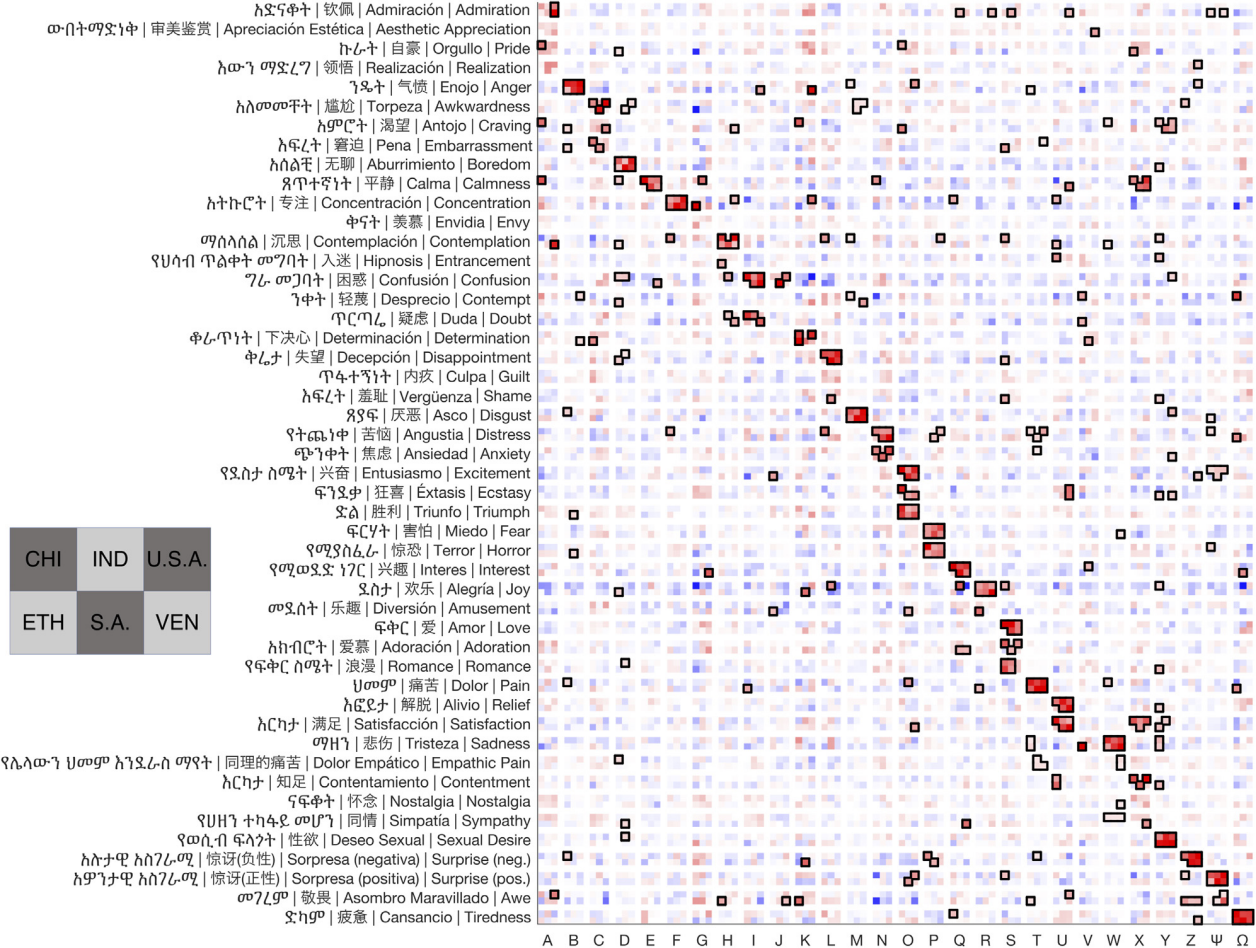
Dimensions of facial expression that emerged as having distinct meanings within or across cultures The meaning of the 28 dimensions of facial expression that were reliably predicted by the model is captured by loadings on the 48 predicted emotion concepts that people used to judge their own expressions (y axis) in each of the 6 countries. Each rectangle is composed of 6 squares that represent the 6 countries (as indicated in the bottom left corner). Squares with dark outlines reflect statistically significant correlations between human judgments in that country and DNN model annotations. Recall that the model was trained to predict judgments in each country (and language) separately. Thus, when multiple countries share statistically significant loadings on similar concepts, it indicates that the dimension of facial expression has a similar meaning across the countries.

Using this method, we uncovered 28 significant dimensions of facial expression that were reliably associated with distinct meanings (Figure 2). More precisely, each of the 28 dimensions corresponds to a pattern of facial movement that is reliably associated with a distinct set of emotion concepts in at least one country or language. In keeping with recent emotion recognition studies,^37^^,^^38^ some facial expressions were found to have shared meanings across all six countries. For instance, the facial expressions corresponding to 12 different emotion concepts in English—”anger,” “boredom,” “concentration,” “disgust,” “fear,” “joy,” “pain,” “sadness,” “sexual desire,” “surprise (positive),” “tiredness,” and “triumph”—were categorized with what we had previously determined were their most direct translations across all six countries and four languages. In four other cases, the correspondence was not exact, but very close: expressions that were associated with “contemplation” in some countries were associated with “doubt” in others, as was the case with “love” and “romance,” “satisfaction” and “contentment,” and “surprise (negative)” and the closest Amharic translation of “awe” (መገረም, which is close in meaning to “surprise” in English). For another five dimensions—“calmness,” “confusion,” “disappointment,” “distress,” and “interest”—loadings were consistent in all six countries but not statistically significant in Ethiopia. Thus, a total of 21 dimensions of facial expression showed a high degree of cultural universality in meaning across the 6 countries.

**Figure 3:**
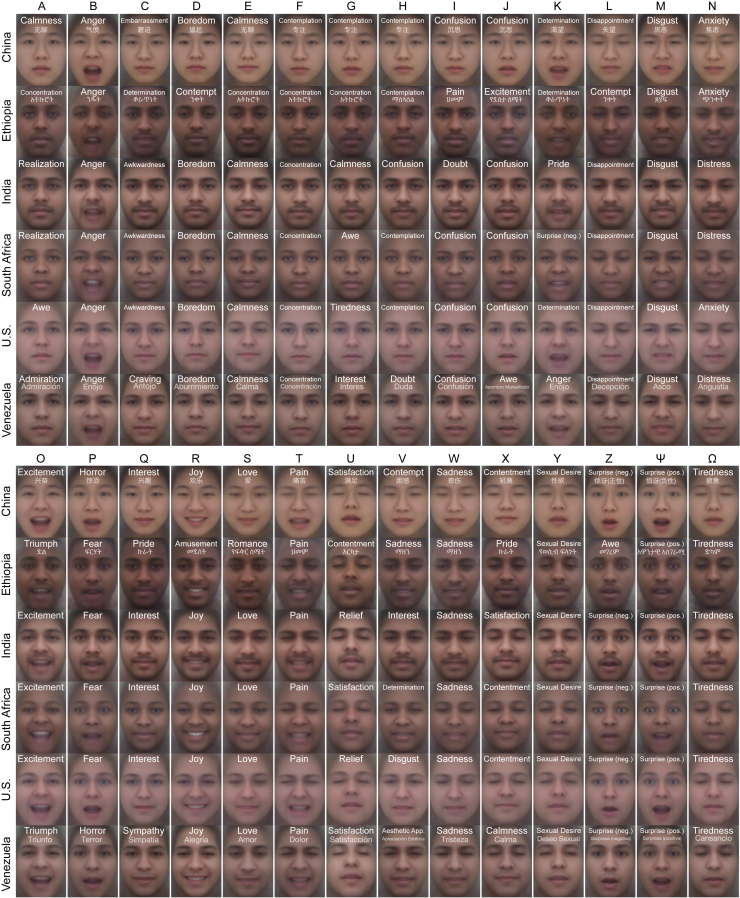
Visualizing the structural dimensions of facial expression that emerged as having distinct meanings within or across cultures We visualized the average facial movements associated with each of the 28 dimensions by morphing together representative mimics in each culture (see STAR Methods for more information). Each of the 28 dimensions are labeled by a letter (A-Z, Ψ, and Ω). The emotion concept that loaded most heavily on each dimension in each country is overlaid on the corresponding image. There are some cases where the same emotion is inferred from multiple facial expressions. Our approach demonstrates that there is not a one-to-one mapping between facial actions and specific emotions, but that there are some cases where the same facial expression could convey different emotions to different individuals or cultures, or some cases where multiple facial expressions could be interpreted as conveying the same emotion.

For some dimensions of facial expression, we found subtle cultural differences in meaning. The expression associated with “awkwardness” in three countries was associated with “determination” in Ethiopia and “craving” in Venezuela. The expression associated with “determination” in three countries was associated with “anger” and “joy” elsewhere. A dimension associated with “calmness” and “satisfaction” in most countries (“Y” in Figures 2 and 3) was associated with “realization” in Ethiopia. There were stronger cultural differences in the meaning of the remaining four dimensions (“A,” “G,” “J,” and “V”). These findings are summarized in Figures 2 and 3.

In total, the 28 dimensions of facial expression— facial movements found to have reliable meanings in at least one country—were 63% preserved in both meaning and translation across the 6 countries (r = 0.80, r^2^ = 0.63, countrywise dimension loadings explained by the average loading), leaving the remaining 37% to be accounted for by either differences in meaning across cultures, imperfect translation across languages, and sampling error (see Figure S4 for breakdown by dimension and country). Where we observed cultural differences, we can see that the facial movements were still imitated very similarly across cultures, confirming that our findings reflected differences in the meanings attributed to the underlying facial movements rather than in the ability to perceive or produce a given set of facial movements.

**Figure 4:**
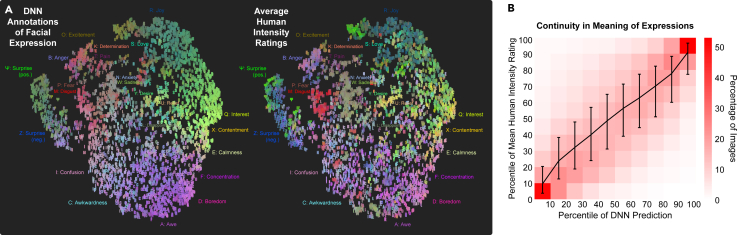
Distribution of facial expressions along the 28 dimensions found to have distinct meanings within or across cultures (A) We used t-distributed stochastic neighbor embedding (t-SNE, see STAR Methods) to visualize the distribution of emotion ratings along the 28 structural dimensions of facial expression that we found to have distinct shared or culture-specific meanings. Projected DNN annotations are shown to the left, and projected average human intensity ratings are shown to the right (for visualization purposes; note that our main analyses did not average across all countries). (B) Comparison between DNN predictions and mean human intensity ratings reveals continuity in the meaning of expressions (x axis = percentile of DNN prediction; y axis = percentile of mean human intensity ratings; error bars = middle 50th percentile). As the intensity of the expression, measured using the DNN, shifted from minimum to maximum along any given dimension (x axis), the meaning perceived in the expression varied smoothly. Critically, expressions lying in the middle of the dimension were not more ambiguous, perceived one way or the other, but were rather perceived reliably as blends or gradations of expressions (i.e., the standard deviation in intensity ratings is not significantly higher around the 50th percentile than around the 25th or 75th).

Finally, as observed in many previous studies,^31^^,^^36^^,^^48^ the emotions attributed to facial movements were not discrete in terms of clear boundaries between categories, but were heterogeneous and varied, reflecting continuous blends of meaning that were reliably associated with subtle combinations of facial movement (Figure 4).

## Discussion

Facial expression is central to how we understand emotion in ourselves and in others, and has been central to scientific debates about emotion.^3^^,^^7^^,^^9^^,^^10^ Contemporary theories of emotion acknowledge that facial expressions are complex and nuanced.^8^^,^^9^ Methodologically, however, theoretical claims have largely rested on the results of small-scale studies that seek to confirm or refute a set of narrow and strong claims about the universality of a small number of facial expressions. This has led to contrasting interpretations of the same data, broad theoretical debate, and a limited understanding of what facial expressions mean to the people making them. To determine what information the face conveys, we combined large-scale cross-cultural data, experimental control to address perceptual biases, and inductive statistical methods.

We performed a large-scale controlled mimicry-based study of what facial expressions mean to tens of thousands of people making them in six countries, and generated data suitable for both machine learning and psychological inference. A DNN tasked with predicting the culture-specific meanings people attributed to their own facial movements while disregarding physical appearance and context discovered 28 dimensions of facial expression with distinct meanings. These dimensions of facial expression were 63% preserved in meaning across the six countries and four languages we studied, with 21 dimensions showing a high degree of universality and the remainder showing subtle to moderate cultural specificity. This is not an exhaustive catalog or taxonomy of distinct emotion concepts or anatomically distinct facial expressions, but the most data intensive, comprehensive dimensional taxonomy of the distinct meanings that facial expressions can reliably convey in a wide range of countries. This work converges with other recent approaches to leverage recent advances in deep learning and affective computing to address fundamental questions about the meaning of facial expressions.^49^^,^^50^^,^^51^

The present study employed an experimental approach to address the limitations of previous large-scale studies of human facial expression. Perceptual ratings of naturally occurring facial expressions are generally confounded by the physical appearance and context of the person making the expression. Here, experimental randomization and mimicry were used to decouple facial movements from these confounds. In addition, previous algorithms for measuring facial expression have been trained on ratings in a single culture using predetermined taxonomies of expression, and have captured a more limited range of facial expressions.^7^^,^^19^ In the present study, we discovered a broader set of dimensions of facial movement that reliably mapped to culture-specific meanings, and used a deeply inductive approach to explore how these meanings converge and diverge across cultures.

### Limitations of the study

This study is not without its limitations. The 28 dimensions of facial expression that we discovered are not exhaustive. The 4,659 facial expressions that participants imitated came from a wide range of datasets encompassing both posed and spontaneous expressions across many contexts and cultures,^7^^,^^26^^,^^27^ but do not capture all possible configurations of facial muscle movement. While the mimic images differed from each other and from the seeds in important ways, it is also true that all images came from similar contexts in that they were all taken by participants sitting at a computer. Certain expressions might show more variability, or might be interpreted differently, in more variable contexts such as social interactions, and more variability in context could also result in more heterogeneous judgments across cultures. Moreover, the concepts participants relied upon to describe the meaning of the expressions—despite encompassing the widest range of distinct emotions for which there is evidence, to our knowledge, across any modality of emotional behavior,^35^ and having previously been found to explain attributions of valence, arousal, and a wide range of other proposed dimensions of appraisal and motivation^7^—may still omit other nuanced meanings that facial expressions can potentially convey, including more nuanced or situational descriptions that could vary by culture.

Most importantly, although the four languages included in the present study are spoken by approximately 40% of the world population (by 25% as a first language), there are 189 other countries and approximately 6,500 other languages that we did not study, across which there are almost certainly other culture-specific facial expressions and meanings. Further work is also needed to examine how facial expression varies as a function of gender, social class, and other sources of individual identity and variation (e.g., see ref. 40). Our hope is that the approach of the present study can accelerate the development of more nuanced taxonomies of facial expression and signaling behavior more generally, giving rise to more sophisticated tools for measuring facial movement and other behavioral dimensions of emotion.

Critiques of survey-based studies conducted in English have expressed concerns about whether English-language emotion concepts have direct translations into other languages and the implications for computational models trained only on English-language annotations.^52^ Importantly, the 28 dimensions of facial expression that we discovered do not depend on any mapping of emotion concepts across languages. We used translations across languages solely to quantify the similarity across cultures in the meaning of the extracted dimensions, but this analysis did not attempt to separate cultural differences in the meaning of facial expressions from shortcomings in the translations of the words used to conceptualize these meanings. Thus, where we observed apparent cultural differences, further work is needed to disentangle differences in meaning from shortcomings in translation. It is also important to note, as a point of interpretation, that the purpose of this study was to understand the inferred meanings of facial expressions, inclusive of both emotions and other emotion-adjacent states. Thus, we use the terminology “emotions and mental states” throughout the article. Further work would be necessary to disentangle which facial expressions are associated with emotion specifically.

### Conclusion

Our results are in keeping with semantic space theory (SST), which conceives of emotions as dimensions of a high-dimensional, continuous state space that explains the systematic variation in emotion-related physiology and behavior.^34^ Consistent with SST, facial expressions were found to be (a) high-dimensional, with nuanced similarities across cultures that could not be reduced to a small number of dimensions (Figures 2 and 3), and (b) continuous, with smooth gradients that corresponded to smooth variations in meaning (Figure 4). The 21 out of 28 dimensions of facial expression for which we observed a particularly high degree of similarity in meaning across cultures—“anger,” “boredom,” “calmness,” “concentration,” “contemplation/doubt,” “confusion,” “disappointment,” “disgust,” “distress,” “fear,” “interest,” “joy,” “love/romance,” “pain,” “sadness,” “satisfaction/contentment,” “sexual desire,” “surprise (negative),” “surprise (positive),” “tiredness,” and “triumph”—encompass 13 out of 16 dimensions of facial expression previously found to occur in similar contexts around the modern world^11^ and 4 out of 5 found to be depicted in consistent contexts in ancient American sculptures,^33^ providing further evidence that a subset of facial expressions may be universal in meaning. These dimensions overlap with those previously found to have shared meanings between cultures using more traditional methods.^53^^,^^54^^,^^55^^,^^56^ They also largely overlap with dimensions of facial expression associated with distinct reported emotional experiences^26^ and with dimensions of evoked emotional experience previously found to correspond to distinctive modes of brain activity in highly interconnected brain regions.^57^ Together, these findings support the central tenet of SST: that one’s emotional state can be described as a position along continuous dimensions of experienced and expressed emotion, and cannot be reduced to low-dimensional taxonomies as traditionally assumed.^34^

## STAR★Methods

### Key resources table

**Table:** 

REAGENT or RESOURCE	SOURCE	IDENTIFIER
**Deposited data**		

Emotion and mental state ratings	This paper	Database: https://doi.org/10.5281/zenodo.8302299

**Software and algorithms**		

Python (version 3.9.5)	Python Software Foundation	https://www.python.org/
Pandas (version 1.2.4)	Python package	RRID: SCR_018214; https://pandas.pydata.org/
NumPy (version 1.20.3)	Python package	RRID: SCR_008633; https://numpy.org
SciPy (version 1.6.3)	Python package	RRID: SCR_008058; https://scipy.org
PyTorch (version 1.8.1)	Python package	RRID: SCR_018536; https://pytorch.org/
MATLAB (version 2021a)	MathWorks	https://matlab.mathworks.com

### Resource availability

#### Lead contact

Further information and requests can be directed to Dr. Jeffrey Brooks (jeff@hume.ai).

#### Materials availability

The data (images) associated with this manuscript are available for non-commercial use upon reasonable request to the corresponding authors.

#### Data and code availability

The raw data associated with this manuscript are available for non-commercial use upon reasonable request to the corresponding authors. Summary data and code associated with this study, including the functions to perform PPCA, are available in the following Zenodo repository: https://zenodo.org/record/8302299.

### Experimental model and study participant details

Participants from China (n = 602; 371 female), Ethiopia (n = 149; 26 female), India (n = 478, 74 female), South Africa (n = 2,131; 970 female), the United States (n = 2,576; 1,346 female), and Venezuela (n = 344; 110 female) were recruited for the mimicry phase of the experiment via psychology recruitment email lists compiled by the authors, survey companies (Node Survey Solutions), and via a range of crowdsourcing platforms (Amazon Mechanical Turk, Clickworker, Prolific, Microworkers, and RapidWorker). On each trial, participants saw a seed facial expression image and were instructed to use their computer webcam to photograph themselves mimicking the expression in the image such that their imitation would be rated similarly to the original image. Participants completed 30 trials per survey and could complete multiple versions of the survey, up to 10 depending on country. Data collection was completed between April, 2021 and December, 2021.

The seed images shown to participants were 4,659 images of people forming facial expressions, extracted from naturalistic datasets of emotional stimuli originating from a diverse range of unspecified cultures (n = 1,166 from ref.^26^), expressive behavior found online using hundreds of search queries for emotion and mental state concepts and emotional contexts (n = 1,500 from ref.^7^; n = 286 from search engines exclusively in China using Chinese translations of the search terms from ref.^7^), and responses to 1,707 emotionally evocative films (n = 1,707 still frames extracted from participants’ responses in ref.^27^).

All images gathered during the mimicry phase of the experiment were used as stimuli in the rating-only phase of the experiment. Participants in the rating-only phase of the experiment [China (*n* = 542; 349 female), Ethiopia (*n* = 78; 18 female), India (*n* = 1,101; 507 female), South Africa (*n* = 2,465; 1,565 female), the United States (*n* = 3,419; 1,983 female), and Venezuela (*n* = 352; 118 female)] were given the option of responding that no face was visible in the image.

All participants provided informed consent and all aspects of the study design and procedure were approved by Heartland IRB (HIRB project no. 031221–315).

### Method details

#### Principal preserved components analysis (PPCA)

PPCA seeks to identify shared dimensions in the latent structure of two datasets measuring the same attributes. Like more established methods such as partial least-squares correlation analysis (PLSC) and canonical correlation analysis (CCA), PPCA examines the cross-covariance between datasets rather than the variance–covariance matrix within a single dataset. However, whereas PLSC and CCA derive two sets of latent variables, α and β, maximizing Cov(Xαi,Yβi] or Corr[Xαi,Yβi], PPCA derives only one variable: α. The goal is to find dimensions of perceived emotion that reliably co-vary across both datasets X and Y.

For an extended validation of the method including a mathematical proof, please refer to ref.^31^

#### Generalized PPCA

Given our present dataset measuring the same attributes comes from six different cultures, we developed a generalized version of the PPCA algorithm (G-PPCA) that extracts linear combinations of attributes that maximally co-vary across multiple datasets (in this case, emotion judgments from six countries). In particular, G-PPCA maximizes the objective function Sum(Cov(α∗X,α∗Y) for X,Y in S) where S is the set of all possible pairwise combinations of datasets. The resulting components are ordered in terms of their level of positive covariance across all six datasets.

We iteratively applied G-PPCA in a leave-one-stimulus-out manner to extract components from the judgments of all but one stimulus, and then projected each country’s ratings of the left-out stimulus onto the extracted components, resulting in cross-validated component scores for each country and stimulus.

#### Facial expression machine learning architecture

Machine-learned representations of facial expressions from the main task were extracted from a deep neural network (DNN) architecture that utilized layers from the FaceNet Inception Resnet v1 model,^44^ pretrained on the VGGFace2 dataset (transfer learning^45^^,^^46^). We froze all layers up until the last convolutional block and unfroze the last convolutional block. On top of this architecture we added the following fresh untrained layers: 2D adaptive average pool (output_size = 1; https://pytorch.org/docs/stable/generated/torch.nn.AdaptiveAvgPool2d.html), followed by a dropout layer (p = 0.6). The features were then flattened and fed to a linear (1790 in features → 512 out features) layer, followed by Batchnorm1d (eps=0.001, momentum = 0.1, affine = True) and a final linear layer (512 in features → 48 ∗ 4 out features).

#### DNN predictions

The DNN was trained on outputs reflecting the average face judgments for each emotion and mental state concept in each language. As discussed in the main text, methodological critiques have questioned the utility of models trained on annotations that only come from the English language or only from English-language speakers. While our main task sourced highly diverse ratings from 6 different countries in 4 different languages, the PPCA and G-PPCA analyses used to establish the preserved dimensions assume that the same 48 concepts are measured in each case (i.e., assuming that the English word “Anger” and the Spanish word “Enojo” represent the same concept). By contrast, the outputs predicted by the DNN were 288 labels comprising the 48 emotion categories rated in six countries and translated as appropriate into the languages Amharic (Ethiopia), Mandarin Chinese (China), English (U.S., South Africa, India), and Spanish (Venezuela).

For each seed image, the target values used during training were the average perceptual judgments in each language for the seed image and mimics of the seed image.

#### Model training

The model was trained on 452,783 participant-generated images of facial expressions drawn from the mimic portion of the main task (i.e., each image was of a participant mimicking a seed image). On a per-country basis there were 59,951 images from China; 29,169 from Ethiopia; 53,830 from India; 160,682 from the United States; 104,473 from South Africa; and 44,337 from Venezuela.

Each sample was associated with a 288-dimensional vector of label-rating pairs (6 countries x 48 emotions). For each seed image, we computed the average of all of the intensity ratings assigned to all mimics of that seed image (both self-report and perceiver ratings of the mimic images) within each country. These averages were scaled to be in the range [0,1] for each emotion concept in each country. We applied this procedure per country and seed. During model training, we assigned the 6x48 label vector of averages of each seed to each mimic image of the respective seed (regardless of the country of the mimic, thus forcing the model to predict what the mimicked facial movements mean to people in each country while ignoring ethnicity). On a per-country basis the ratings were averaged from a total of 130,298 ratings from China; 39,592 from Ethiopia; 158,760 from India; 287,830 from South Africa; 372,625 from the United States, and 95,396 from Venezuela.

All images were cropped, resized to a 160x160 pixel square centered around the face using MTCNN^47^ (as was the original input to the trained FaceNet architecture), and pre-whitened. The minimum face size to search for in an image was 20x20 pixels. A random 95% of the mimic images were used for training (with the remainder reserved for informal evaluation and unused in the present paper).

Training was completed in two epochs with the following hyperparameters: batch size = 1024; mean-squared error (MSE) loss function; ADAM optimizer (lr=1eˆ-4, weight decay=1eˆ-4); learning rate scheduler gamma = 0.1; step size = 20 batches. MSE did not improve with continued training.

#### Dimension morphs

We visualized the perceptual dimensions of the data by morphing together representative faces along each dimension using the OpenCV library in Python. Specifically, we first computed the locations of 68 facial landmarks in each image, then performed a Delaunay triangulation of the landmark locations. Finally, we performed affine transformations to project the pixels within each triangle of the Delaunay triangulation of each face onto the average facial landmark coordinates of the faces being morphed and averaged the resulting pixel values across all faces. This procedure was used to create a composite image of representative mimic images from each culture by averaging all mimics of the 5 seed images scoring maximally on each dimension and the 5 seed images with the highest differential scores (maximum score minus the second highest score) on each dimension.

### Quantification and statistical analysis

#### Significance testing

To determine the statistical significance of each component extracted from the G-PPCA analysis, we sought to ensure that the extracted dimensions not only reflect shared structure preserved across all six countries, but also reflect significantly preserved dimensions across pairs of countries in the analysis. First, we calculated the partial Pearson correlation between corresponding component scores for each country pair, iteratively partialling out each previous component, and calculating statistical significance separately for each dimension. Within country pairs, *p-*values for the 48 dimensions were FDR-corrected using the Benjamini-Hochberg procedure.^58^

Group-level statistical significance of the generalized dimensions was determined by representing each dimension as a graph with countries as nodes and binary statistical significance ( *p* < .05, FDR-corrected) as edges. We then retained dimensions whose statistical significance graph was unipartite (that is, could not be partitioned - in this case meaning that for any given dimension the component scores for any given country were significantly positively correlated with the corresponding component scores for at least two other countries).

#### Factor rotation

To interpret and visualize the preserved components, we applied Varimax factor rotation. Varimax is a factor rotation method that minimizes the number of factors needed to explain a variable, simplifying the structure of the factor matrix and making dimensions more interpretable.^59^ We employed an implementation of Varimax available in Python’s statsmodels package (https://www.statsmodels.org/stable/generated/statsmodels.multivariate.factor_rotation.rotate_factors.html).

#### t-distributed stochastic neighbor embedding (t-SNE)

We also sought to establish and visualize how the dimensions of facial emotion perception are distributed. As in previous work, we approached this by visualizing the data using t-distributed stochastic neighbor embedding (t-SNE^60^). This method projects high-dimensional data onto two nonlinear axes, such that the local distances between data points are accurately preserved while more distinct data points are separated by longer, more approximate, distances.

#### Extracting significant DNN output dimensions

To identify dimensions of facial expression captured by the DNN that were reliably associated with distinct meanings in one or more cultures, we applied PPCA between the 288 outputs of the DNN applied to the seed images, which directly measure facial movement in the seed images, and the 288 averaged perceptual judgments of the seed images (ratings of 48 concepts in each of six countries). This analysis captures the dimensions along which country-specific perceptual judgments of naturalistic facial expressions are influenced by facial movement.

To assess the significance of the dimensions extracted using PPCA, we used a leave-one-out cross-validation method. Specifically, we iteratively performed PPCA between the DNN outputs and the averaged perceptual judgments of all but one of the seed images and computed the scores of each dimension extracted by PPCA on the DNN outputs and averaged perceptual judgments of the held-out images. Finally, we concatenated and correlated the PPCA scores of the held-out DNN outputs and judgments. To control for non-linear monotonic dependencies between extracted dimensions, we used partial Spearman correlations, where for each PPCA dimension we controlled for the PPCA scores on all previous dimensions. To determine the significance of each dimension, we used a bootstrapping method, iteratively repeating the correlation procedure while randomly resampling the seed images (1000 iterations with replacement). P-values were taken as one minus the proportion of times that the correlation exceeded zero across resampling iterations.

After computing p-values, we used a conservative method of correction for false discovery rate (FDR) that combined multiple FDR-correction methods. Specifically, we used Benjamini-Hochberg^58^ FDR correction across the first 48 PPCA dimensions (as we were interested in variations of 48 potentially distinct emotion concepts and their translations across countries) at an alpha of .05. We also separately performed a ForwardStop sequential FDR correction procedure.^61^ Finally, we determined the signal-to-noise ratio (SNR) of the correlations corresponding to each PCA dimension (the correlation divided by the standard deviation computed using bootstrapping [see above]) and applied a threshold of 3 to the SNR to extract more stable dimensions. We only kept dimensions that met all three of these criteria. We applied factor rotation using the varimax criterion^59^ to these dimensions.

To assess the significance of the individual loadings of emotion concepts on the extracted dimensions, we used a bootstrapping method. Specifically, we performed the entire PPCA analysis repeatedly after resampling the seed images with replacement, extracting the significant dimensions and performing factor analysis each time. For each dimension, we then tested the significance of the top N loadings, with N varying from 1 to 288, by determining how often, across resampling the iterations, there existed a dimension with all of these top N loadings pointing in the same direction. This estimates the proportion of times a dimension with these coloadings would be extracted if we repeated the entire study. We took one minus this proportion as the p-value. As N varies from 1 to 288, the p-value can only increase because more loadings are included in the test (and therefore the probability of all loadings pointing in the same direction decreases monotonically). For each dimension, we applied a ForwardStop FDR-correction procedure^61^ at an alpha of .05 to determine the number of significant loadings.

## Supplemental information

Document S1. Figures S1‒S4 and Tables S1 and S2
