# Supplementary material for: Deep learning reveals what facial expressions mean to people in different cultures
Source: iScience. 2024 Feb 10;27(3):109175. doi: 10.1016/j.isci.2024.109175 (PMC10906517; doi:10.1016/j.isci.2024.109175)
Supplement: Document S1. Figures S1‒S4 and Tables S1 and S2 [file mmc1.pdf]

iScience, Volume *27*

Supplemental information

Deep learning reveals what facial expressions mean to people in different cultures

Jeffrey A. Brooks, Lauren Kim, Michael Opara, Dacher Keltner, Xia Fang, Maria Monroy, Rebecca Corona, Panagiotis Tzirakis, Alice Baird, Jacob Metrick, Nolawi Taddesse, Kiflom Zegeye, and Alan S. Cowen

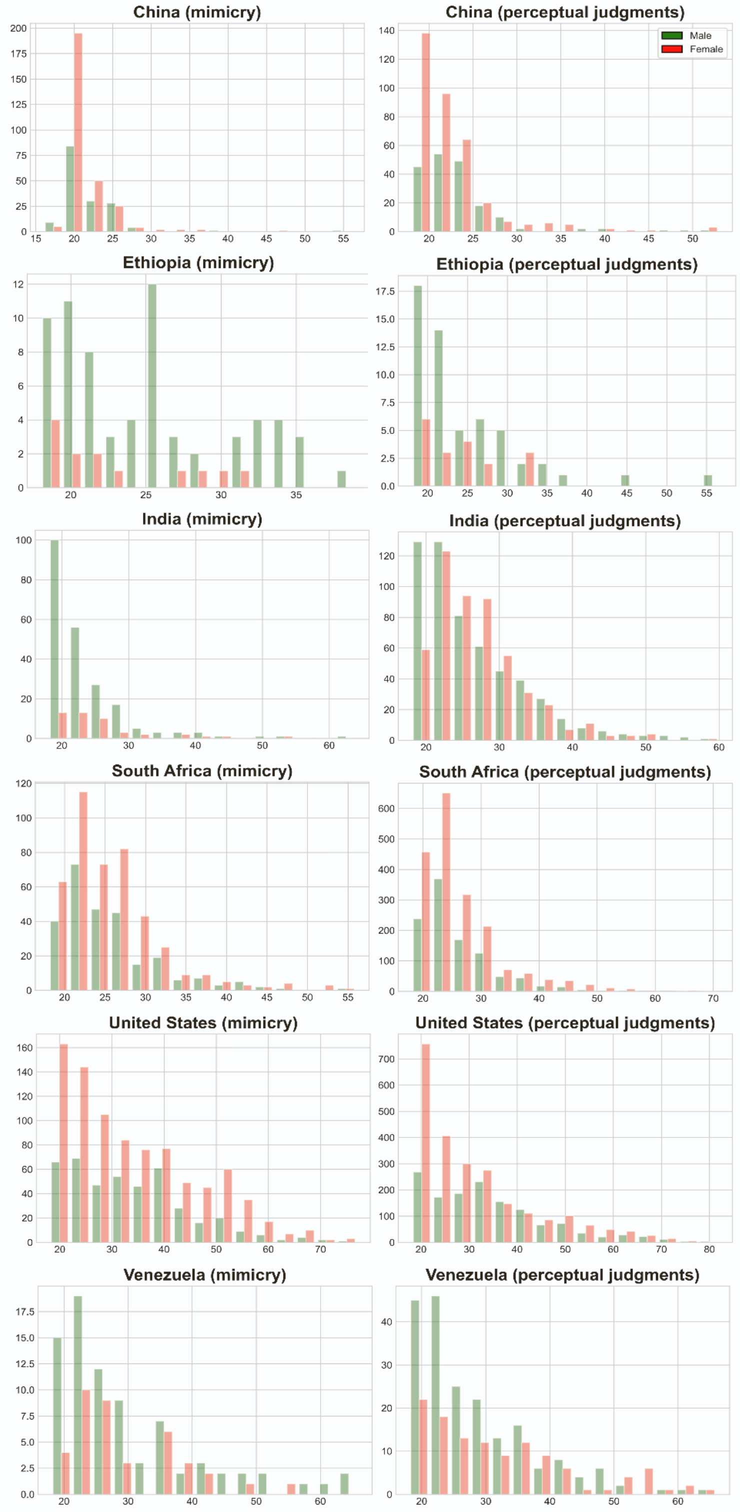

Fig. S1. Age and gender distributions for each sample within each country. (Related to STAR Methods).

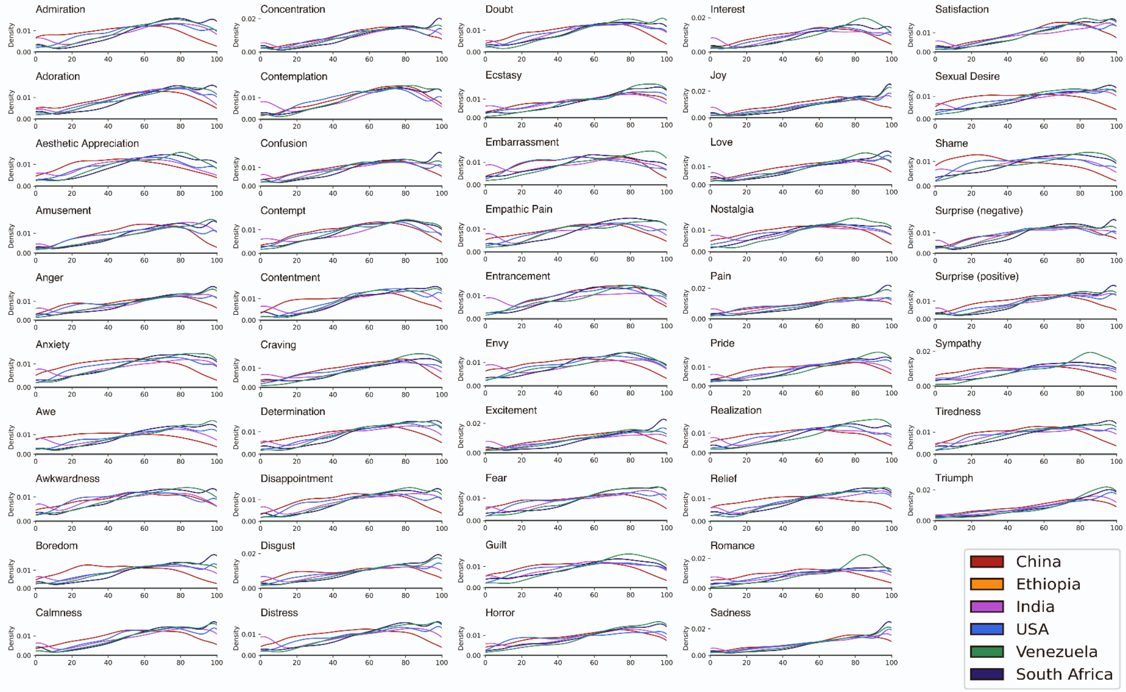

Fig. S2. Density plots of intensity ratings for each emotion and mental state term. (Related to Figures 2-4). Distributions of judgments are shown for each emotion and mental state concept. Each country's ratings are shown on a different line. On each trial, participants were asked to judge what they thought the person was feeling by selecting from 48 terms for emotions and mental states and then rating each selection from 1-100, with values reflecting the perceived intensity of the emotion or mental state. Participants were required to select a value on a rating scale for at least one category. English terms were used in the three out of six countries where English is an official language (India, South Africa, and the United States). In China, ratings were collected in Chinese; in Ethiopia, ratings were collected in Amharic; and in Venezuela, ratings were collected in Spanish.

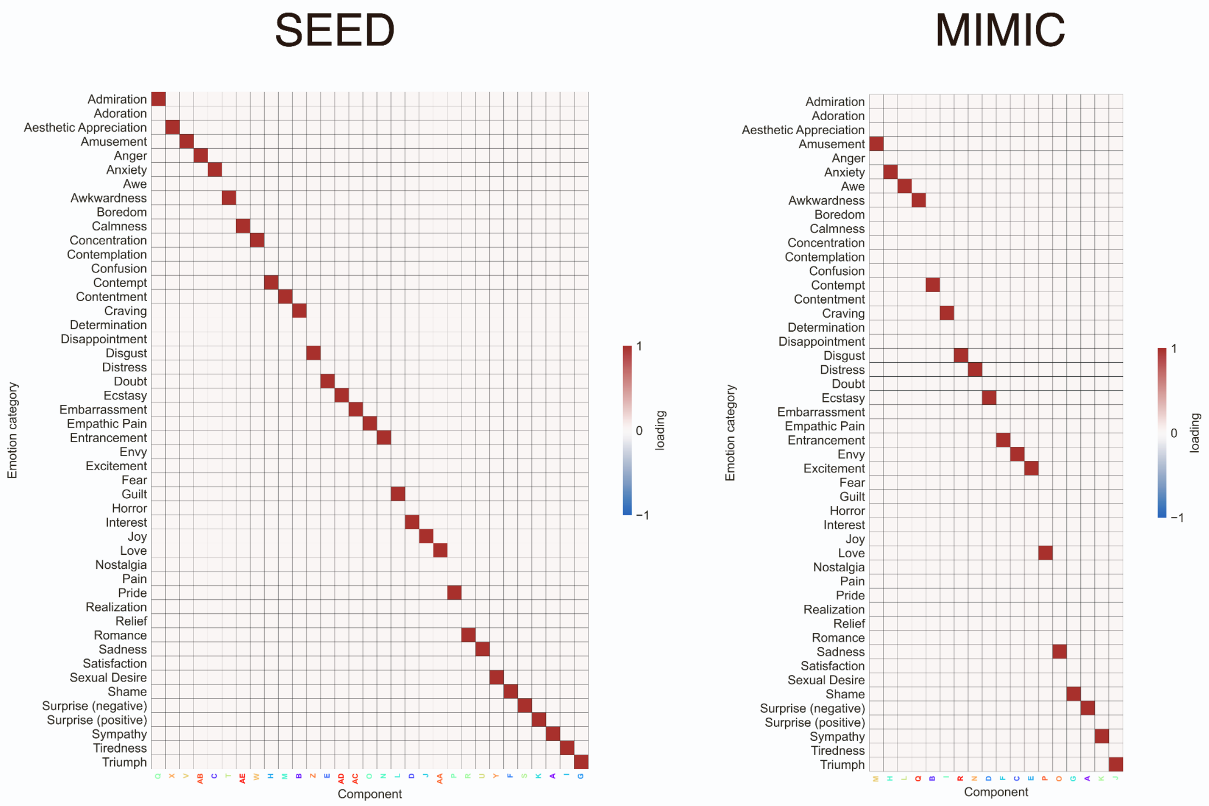

Fig. S3. Generalized PPCA (G-PPCA) Results. (Related to Figure 2). G-PPCA extracts linear combinations of attributes that maximally co-vary across three or more datasets (in this case, emotion and mental state judgments from 6 countries). The resulting components are ordered in terms of their level of positive covariance across all 6 datasets. We ran G-PPCA on judgments of facial expressions, both from the mimicry phase (in which participants provided judgments of the seed images) and the rating-only phase (in which participants provided judgments of the mimic images produced during the mimicry phase). G-PPCA was conducted on a per-seed basis in both cases. Specifically, inputs to G-PPCA for the mimicry phase were average judgments of the seed images and inputs to G-PPCA for the rating-only phase were average judgments of the mimic images for each seed. We found that 31 dimensions were preserved in judgments of the seed images, and 18 dimensions were preserved in judgments of the mimic images across cultures.

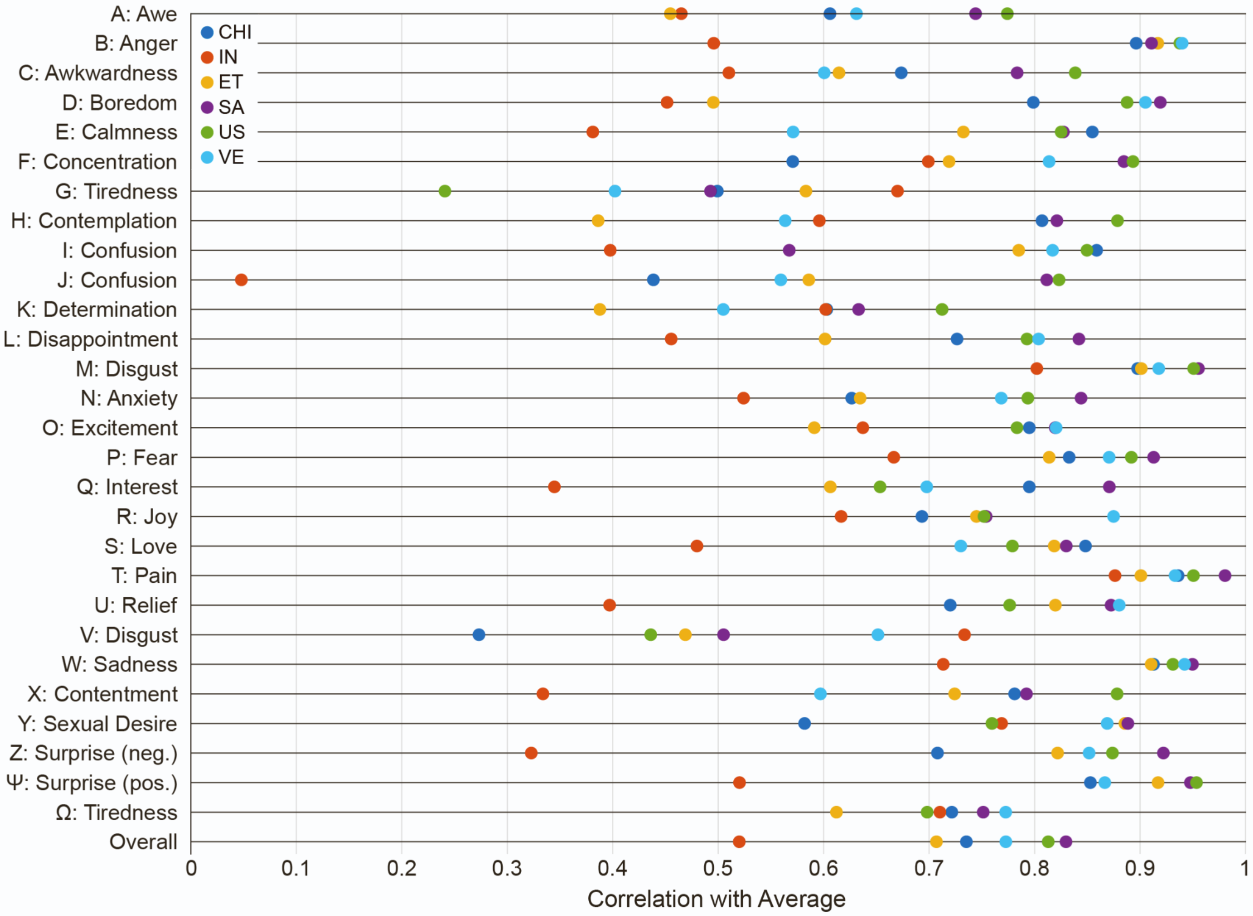

Fig. S4. Loading correlation by country and dimension. (Related to Figures 2-4). Loading correlations are depicted separately for each country and dimension. In total, the 28 dimensions of facial expression— facial movements found to have reliable meanings in at least one country—were 58% preserved in both meaning and translation across the 6 countries (r = .76, r^2 = .58, countrywise dimension loadings explained by the average loading).

Table S1. Extended demographic information. (Related to STAR Methods).

	Sample Size		Images By Gender			By Age			By Race/Ethnicity					Number of Ratings		
**Country**	Unique Subjects	# Images	Male	Female	Non-binary	10th %	Avg.	90th %	White	Black	Asian	Hispanic	Other	Self-report	Perceptual	Total
United States	8,492	170,013	71,671	89,446	8,896	20	34.3	53	117,620	23,037	12,897	9,588	4,659	170,013	202,612	372,625
Venezuela	744	47,734	30,930	16,644	160	20	28.6	42	0	0	0	47,734	0	47,734	60,703	108,437
Ethiopia	1,212	29,773	20,837	6,012	21	19	25.3	31	0	29,773	0	0	0	29,773	10,173	39,946
China	1,165	60,498	22,467	37,508	524	19	21.7	25	0	0	60,498	0	0	60,499	69,556	130,055
India	1,562	58,054	48,437	9,526	91	19	25.5	35	386	0	57,458	90	120	58,054	103,398	161,452
South Africa	6,481	107,364	38,934	67,066	1,364	20	26.4	35	12,032	82,090	2,540	30	9,702	107,364	166,207	273,571
Total	*19,656*	***473,436***	*233,276*	*226,202*	*11,056*	*19.6*	*27.0*	*36.6*	*130,038*	*134,900*	*133,393*	*57,442*	*14,481*	*473,437*	*612,649*	***1,086,086***

Table S2. All 48 emotion terms used in each language. (Related to Figure 2)

Amharic	Chinese	English	Spanish
አድናቆት	钦佩	Admiration	Admiración
አክብሮት	爱慕	Adoration	Adoración
ውበትማድነቅ	审美鉴赏	Aesthetic appreciation	Apreciación Estética
መደሰት	乐趣	Amusement	Diversión
ንዴት	气愤	Anger	Enojo
ጭንቀት	焦虑	Anxiety	Ansiedad
መገረም	敬畏	Awe	Asombro Maravillado
አለመመቸት	尴尬	Awkwardness	Torpeza
አሰልቺ	无聊	Boredom	Aburrimiento
ጸጥተኛነት	平静	Calmness	Calma
አትኩሮት	专注	Concentration	Concentración
ማሰላሰል	沉思	Contemplation	Contemplación
ግራ መጋባት	困惑	Confusion	Confusión
ንቀት	轻蔑	Contempt	Desprecio
እርካታ	知足	Contentment	Contentamiento
አምሮት	渴望	Craving	Antojo
ቆራጥነት	下决心	Determination	Determinación
ቅሬታ	失望	Disappointment	Decepción
ጸያፍ	厌恶	Disgust	Asco
የትጨነቀ	苦恼	Distress	Angustia
ጥርጣሬ	疑虑	Doubt	Duda
ፍንደቃ	狂喜	Ecstasy	Éxtasis
እፍረት	窘迫	Embarrassment	Pena
የሌላውን ህመም እንደራስ ማየት	同理的痛苦	Empathic Pain	Dolor Empático
የህሳብ ጥልቀት መግባት	入迷	Entrancement	Hipnosis
ቅናት	羡慕	Envy	Envidia
የደስታ ስሜት	兴奋	Excitement	Entusiasmo
ፍርሃት	害怕	Fear	Miedo
ጥፋተኝነት	内疚	Guilt	Culpa
የሚያስፈራ	惊恐	Horror	Terror
የሚወደድ ነገር	兴趣	Interest	Interes
ደስታ	欢乐	Joy	Alegría
ፍቅር	爱	Love	Amor
ናፍቆት	怀念	Nostalgia	Nostalgia
ህመም	痛苦	Pain	Dolor
ኩራት	自豪	Pride	Orgullo
እውን ማድረግ	领悟	Realization	Realización
እፎይታ	解脱	Relief	Alivio
የፍቅር ስሜት	浪漫	Romance	Romance
ማዘን	悲伤	Sadness	Tristeza
የወሲብ ፍላጎት	满足	Satisfaction	Satisfacción
አሉታዊ አስገራሚ	性欲	Sexual Desire	Deseo Sexual
አዎንታዊ አስገራሚ	羞耻	Shame	Vergüenza
የህዘን ተካፋይ መሆን	惊讶(负性)	Surprise (negative)	Sorpresa (negativa)
ድካም	惊讶(正性)	Surprise (positive)	Sorpresa (positiva)
ድል	同情	Sympathy	Simpatía
	疲惫	Tiredness	Cansancio
	胜利	Triumph	Triunfo
